# Propensity score analysis comparing survival between definitive chemoradiotherapy and esophagectomy with adjuvant chemoradiotherapy in patients with esophageal squamous cell carcinoma

**DOI:** 10.1371/journal.pone.0271338

**Published:** 2022-10-13

**Authors:** Yi-Lin Chang, Ya-Fu Cheng, Hui-Shan Chen, Siao-Chi Wu, Wei-Heng Hung, Heng-Chung Chen, Chang-Lun Huang, Ching-Yuan Cheng, Bing-Yen Wang

**Affiliations:** 1 Division of General Surgery, Department of Surgery, Changhua Christian Hospital, Changhua, Taiwan; 2 Division of Thoracic Surgery, Department of Surgery, Changhua Christian Hospital, Changhua, Taiwan; 3 Department of Health Care Administration, Chang Jung Christian University, Tainan, Taiwan; 4 Institute of Health and Welfare Policy, National Yang-Ming University, Taipei, Taiwan; 5 Department of Post-Baccalaureate Medicine, College of Medicine, National Chung Hsing University, Taichung, Taiwan; 6 School of Medicine, Chung Shan Medical University, Taichung, Taiwan; 7 School of Medicine, College of Medicine, Kaohsiung Medical University, Kaohsiung, Taiwan; 8 Institute of Genomics and Bioinformatics, National Chung Hsing University, Taichung, Taiwan; 9 Center for General Education, Ming Dao University, Changhua, Taiwan; Baylor College of Medicine, UNITED STATES

## Abstract

**Introduction:**

The purpose of the current study is to compare definitive chemoradiotherapy and esophagectomy with adjuvant chemoradiotherapy in patients with cT1-3/N0-3 esophageal squamous cell carcinoma in survival.

**Methods:**

Records from 2008 to 2014 of 4931 patients with clinical T1-3/N0-3 esophageal squamous cell carcinoma receiving definitive chemoradiotherapy or esophagectomy with adjuvant chemoradiotherapy were obtained from the Taiwan Cancer Registry. Univariable and multivariable analyses were performed and propensity score matching was used to minimize the bias. Overall survival was compared between definitive chemoradiotherapy and esophagectomy with adjuvant chemoradiotherapy, and also in the three different clinical stages.

**Results:**

Definitive chemoradiotherapy was performed on 4381 patients, and 550 patients received esophagectomy adjuvant chemoradiotherapy. Each group produced 456 patients for comparison after propensity score matching. The 1-year, 2-year, and 3-year overall survival rates for matched patients in with definitive chemoradiotherapy group were 57.18%, 31.92%, and 23.8%. The 1-year, 2-year, and 3-year overall survival rates for matched patients treated in the esophagectomy with adjuvant chemoradiotherapy group were 72.35%, 45.74%, and 34.04%(p<0.0001). In multivariable analysis, treatment modality was an independent prognostic factor. Esophagectomy with adjuvant chemoradiotherapy provided better survival outcome than definitive chemoradiotherapy for patients with clinical stage II/III disease. As for patients with clinical stage I disease, there was no significant survival difference between definitive chemoradiotherapy and esophagectomy with adjuvant chemoradiotherapy.

**Conclusions:**

Esophagectomy with adjuvant chemoradiotherapy provided better survival than definitive chemoradiotherapy in clinical II/III esophageal squamous cell carcinoma. However, more data are needed to conduct a convincing conclusion in clinical stage I patients.

## Introduction

Esophageal squamous cell carcinoma(ESCC) is one of the leading causes of cancer-related death. Esophageal cancer is associated with poor survival outcome because of its aggressive tumor biology, diagnosis at advanced stages, and high recurrence rate [1–3]. ESCC has high prevalence in East Asia, with Taiwan being one of the high-prevalence areas.

Between 2008 and 2014, a total of 14,394 patients were newly diagnosed with ESCC in Taiwan. Most of them received definitive chemoradiotherapy(CRT)(46%), neoadjuvant CRT with esophagectomy(13.6%), esophagectomy alone(10.8%), or esophagectomy with adjuvant CRT(4.8%) [4]. Some head-to-head comparisons between these treatment strategies have been published [4–6]. Our previous studies had compared definitive CRT with neoadjuvant CRT followed by esophagectomy and definitive CRT with esophagectomy alone. Definitive CRT was suggested as a major treatment for locally advanced ESCC by the National Comprehensive Cancer Network(NCCN) guidelines, whereas adjuvant CRT is suggested for patients with positive surgical margins [7]. The role of adjuvant CRT for ESCC patients has not been investigated yet. To date, literature comparing definitive CRT and esophagectomy with adjuvant CRT is unavailable. The aim of this study was to compare the survival difference between definitive CRT and esophagectomy with adjuvant CRT, and propensity score matching was performed to minimize selection bias.

## Methods

This study was designed as a retrospective study, and the number of the IRB is 171116. The patient clinical data were retrieved from the Taiwan Cancer Registry(TCR). The TCR is a national population-based cancer database organized and funded by the Health Promotion Administration, Ministry of Health and Welfare, of the executive branch of the central government. Currently, 80 hospitals are included in this registration that accounts for more than 90% of all cancer cases in Taiwan. The TCR contains information including demographic data(i.e., sex, age, place of residence), stage at initial diagnosis(i.e., clinical and pathological stage), tumor-related characteristics(date of diagnosis, primary site, tumor size, histology, grade/differentiation, modalities used for diagnosis, regional LN examined), treatment modality(date of initial treatment, date of initial surgery, surgical margins, type and course of chemotherapy, course of radiotherapy), and follow-up information(e.g., date of recurrence, last contact or death). The data of this study retrieved from the TCR database required an application, which needs submission every time.

We identified 14,394 patients in Taiwan who were diagnosed between 2008 and 2014 with ESCC. Of those patients, 6614 were treated with definitive CRT and 693 were treated with esophagectomy with adjuvant CRT. Patients received chemotherapy or radiotherapy alone were excluded from the definitive CRT group. The clinical and pathological diagnoses were staged according to 7^th^ edition of the AJCC Cancer Staging Manual [8]. Among those 7307 patients, 5119 patients were TNM stage T1-3 and N0-3. Patients with incomplete clinical stages were excluded, and 4931 patients were finally included in this study.

Propensity score matching between definitive CRT and esophagectomy with adjuvant CRT was performed to minimize the bias resulting from nonrandomized assignment.

### Statistical analysis

Categorical was compared using the chi-sqaure test and continuous variables was compared using the Student’s 2-tailed t test, respectively. The Charlson score was used to adjust the bias of pre-existing comorbidities, and it is a tool for classifying clinical physical comorbidities and risk adjustment in analysis. The overall survival (OS) time was calculated from the date of initial treatment to either the date of death or December 31, 2014. To investigate the treatment effect on OS, the clinical variables: age, sex, Charlson score, clinical T, clinical N, clinical stage, histologic grade, tumor location, tumor length, and treatment modality, were included into univariable analysis and multivariable analysis before and after propensity score matching. The Cox proportional hazards regression model was used for univariable survival analysis and multivariable survival analysis. The OS curves were calculated by the Kaplan-Meier method, and the difference was determined by the log-rank test. Propensity scores were estimated using a logistic model that included the following variables: age, sex, Charlson score, clinical T, clinical N, clinical stage, histologic grade, tumor location, and tumor length. Propensity score matching between definitive CRT and esophagectomy with adjuvant CRT was performed to minimize the bias due to nonrandomized assignment. A p-value < 0.05 was considered statistically significant. SAS software (SAS System for Windows, version 9.3; SAS Institute, Cary, North Carolina) and SPSS software version 20 (IBM, Armonk, New York, USA) were used to perform the statistical analysis.

## Results

A total of 4931 ESCC clinical T1-3 and N0-3 patients undergoing definitive CRT (N = 4381) or esophagectomy with adjuvant CRT (N = 550) between 2008 and 2014 were included in this study. The clinical demographics are summarized in Table 1. The mean ages were 57.8±11.2 in the definitive CRT group and 54.2±9.1 years in the esophagectomy with adjuvant CRT group (p<0.001). There was no significant difference in sex. Patients in the definitive CRT group were more likely to have an older age, a higher Charlson score, a higher clinical T stage, a higher clinical N stage, stage III disease, a tumor located in the upper or middle third of the esophagus, and a longer tumor length compared with patients in the esophagectomy with adjuvant CRT group. From both groups, 456 patients were selected via propensity score matching based on pre-treatment clinical variables. After matching, there was no significant difference between the groups. The well-matched 456 patients in each group are shown in Table 1.

**Table 1:** Clinical demographic data of all ESCC patients treated via definitive CRT or esophagectomy with adjuvant CRT.

Characteristics	All Patients						Propensity-Matched Patients					
	CCRT (N = 4,381)			Surgery with adjuvant therapy (N = 550)		P-value	CCRT (N = 456)			Surgery with adjuvant therapy (N = 456)		P-value
	N	%		N	%		N	%		N	%	
Age (year)												
Mean ± SD	57.8±11.2			54.2±9.1		<0.001	55.3±11.3			54.9±9.1		0.5736
Sex						0.4660						0.8832
Male	4,173	95.25		520	94.55		431	94.52		432	94.74	
Female	208	4.75		30	5.45		25	5.48		24	5.26	
Charlson score	0.8±1.3			0.5±0.9		<0.001	0.7±1.2			0.6±1.0		0.0619
Clinical T						<0.001						0.4134
1 / 2	1,047	23.90		216	39.27		180	39.47		168	36.84	
3	3,334	76.10		334	60.73		276	60.53		288	63.16	
Clinical N						<0.001						0.7296
0	741	16.91		218	39.64		168	36.84		161	35.31	
1	1,843	42.07		269	48.91		233	51.10		235	51.54	
2	1,090	24.88		54	9.82		44	9.65		52	11.40	
3	707	16.14		9	1.64		11	2.41		8	1.75	
Clinical stage						<0.001						0.8599
I	169	3.86		43	7.82		35	7.68		33	7.24	
II	769	17.55		247	44.91		196	42.98		185	40.57	
III	2,192	50.03		235	42.73		204	44.74		215	47.15	
IV	1,251	28.56		25	4.55		21	4.61		23	5.04	
Grade						<0.001						0.9113
Well differentiated / Moderately differentiated	2,033	46.40		351	63.82		308	67.54		302	66.23	
Poorly differentiated/ Undifferentiated	922	21.05		182	33.09		139	30.48		145	31.80	
Unknown	1,426	32.55		17	3.09		9	1.97		9	1.97	
Tumor location						<0.001						0.9959
Lower	957	21.84		183	33.27		132	28.95		129	28.29	
Middle	1,483	33.85		174	31.64		159	34.87		159	34.87	
Upper	985	22.48		44	8.00		41	8.99		42	9.21	
Unknown	956	21.82		149	27.09		124	27.19		126	27.63	
Tumor length (mm)												
Mean ± SD	57.0±29.8			44.5±20.7		<0.001	44.7±24.0			45.5±20.1		0.6055

The 1-year, 2-year and 3-year OS rates in the definitive CRT group before matching were 46.09%, 24.88% and 17.87%. The 1-year, 2-year and 3-year OS rates in the esophagectomy with adjuvant CRT group before matching were 74.35%, 47.81% and 36.02%. The 1-year, 2-year and 3-year OS rates for matched patients in the definitive CRT group were 57.18%, 31.92% and 23.80%. The 1-year, 2-year and 3-year OS rates for matched patients in the esophagectomy with adjuvant CRT group were 72.35%, 45.74% and 34.04%. The 1-year, 2-year and 3-year OS rates were higher in the esophagectomy with adjuvant CRT group compared to definitive CRT group (p<0.001) (Table 2).

**Table 2:** Overall survival rate of all ESCC patients treated via definitive CRT or esophagectomy with adjuvant CRT.

Timing	All Patients			Propensity-Matched Patients		
	CRT (N = 4,381)	Surgery with adjuvant CRT (N = 550)	P-value	CRT (N = 456)	Surgery with adjuvant CRT (N = 456)	P-value
One year	46.09%	74.35%	<0.001	57.18%	72.35%	<0.001
Two year	24.88%	47.81%	<0.001	31.92%	45.74%	<0.001
Three year	17.87%	36.02%	<0.001	23.80%	34.04%	<0.001

Hazards regression analysis identified clinical T3, clinical N+, clinical stage, differentiated grade, tumor location, tumor length and treatment modality as significant prognostic factors before propensity score matching. After matching, clinical T3, clinical N3, clinical stage III/IV, poorly differentiated/undifferentiated, tumor length and treatment modality were significant prognostic factors (Table 3).

**Table 3:** Univariable analysis for all patients before and after propensity score matching.

Characteristics	All Patients			Propensity-Matched Patients		
	HR	95% CI	p-value	HR	95% CI	p-value
Age (year)	1.00	1.00–1.00	0.3120	1.00	1.00–1.01	0.5847
Sex						
Male	1			1		
Female	0.77	0.66–0.90	0.0007	0.82	0.57–1.17	0.2708
Charlson socre	1.01	0.98–1.04	0.4718	1.07	1.00–1.14	0.0623
Clinical T						
1 / 2	1			1		
3	1.61	1.49–1.74	<0.001	1.60	1.36–1.89	<0.001
Clinical N						
0	1			1		
1	1.45	1.33–1.59	<0.001	1.18	1.00–1.39	0.0564
2	1.63	1.47–1.80	<0.001	1.04	0.79–1.38	0.7692
3	2.21	1.98–2.46	<0.001	1.77	1.05–2.98	0.0331
Clinical stage						
I	1			1		
II	1.29	1.07–1.56	0.0090	1.38	0.98–1.93	0.0637
III	1.98	1.65–2.37	<0.001	1.75	1.26–2.45	0.0009
IV	3.19	2.65–3.83	<0.001	2.73	1.74–4.27	<0.001
Grade						
Well differentiated/ Moderately differentiated	1			1		
Poorly differentiated / Undifferentiated	1.11	1.03–1.21	0.0078	1.23	1.04–1.45	0.0132
Unknown	1.17	1.08–1.25	<0.001	0.89	0.50–1.58	0.6936
Tumor location						
Upper	1			1		
Lower	1.15	1.04–1.26	0.0050	1.19	0.88–1.60	0.2600
Middle	1.13	1.04–1.24	0.0057	1.13	0.85–1.52	0.4038
Tumor length	1.01	1.01–1.01	<0.001	1.01	1.00–1.01	<0.001
Treatment modality						
CRT	1			1		
Surgery with adjuvant CRT	0.53	0.47–0.59	<0.001	0.69	0.59–0.80	<0.001

Charlson score, clinical T3/4, clinical stage III/IV, poorly differentiated, undifferentiated, tumor location, and tumor length were associated with worse outcomes before propensity score matching in the multivariable analysis. After matching, Charlson score(Hazard ratio:1.10, 95% CI:1.03–1.18, P = 0.0061), clinical T3/4(HR:1.44, 95% CI:1.12–1.86, P = 0.0049), poorly differentiated/undifferentiated(HR:1.25, 95% CI:1.06–1.48, P = 0.0101), and tumor length(HR:1.00, 95% CI:1.00–1.01, P = 0.0322) were independently associated with worse outcomes, whereas the use of surgery with adjuvant CRT was an independent factor for longer survival (Table 4).

**Table 4:** Multivariable analysis for all patients before and after propensity score matching.

	All Patients			Propensity-Matched Patients		
	AHR	95% CI	p-value	AHR	95% CI	p-value
Age (year)	1.00	1.00–1.00	0.4780	1.00	0.99–1.01	0.6907
Sex						
Male	1			1		
Female	0.81	0.67–0.98	0.0260	0.86	0.60–1.23	0.4120
Charlson score	1.05	1.02–1.08	0.0038	1.10	1.03–1.18	0.0061
Clinical T						
1 / 2	1			1		
3 / 4	1.21	1.08–1.36	0.0014	1.44	1.12–1.86	0.0049
Clinical N						
0	1			1		
1	0.90	0.76–1.07	0.2314	0.91	0.69–1.22	0.5293
2	0.97	0.80–1.18	0.7729	0.80	0.53–1.21	0.2876
3	1.14	0.93–1.40	0.2053	1.33	0.73–2.43	0.3553
Clinical stage						
I	1			1		
II	1.19	0.92–1.53	0.1981	1.22	0.84–1.77	0.2933
III	1.48	1.07–2.05	0.0173	1.32	0.76–2.30	0.3230
IV	2.18	1.59–3.00	<0.001	2.41	1.36–4.28	0.0027
Grade						
Well differentiated/ Moderately differentiated	1			1		
Poorly differentiated / Undifferentiated	1.11	1.00–1.22	0.0496	1.25	1.06–1.48	0.0101
Unknown	1.04	0.95–1.15	0.3644	0.89	0.50–1.60	0.7034
Tumor location						
Upper	1			1		
Lower	1.26	1.12–1.42	0.0002	1.20	0.88–1.63	0.2551
Middle	1.23	1.10–1.37	0.0003	1.29	0.95–1.74	0.1003
Unknown	1.13	1.00–1.28	0.0437	1.23	0.91–1.68	0.1843
Tumor length	1.00	1.00–1.01	<0.001	1.00	1.00–1.01	0.0322
Treatment modality						
CRT	1			1		
Surgery with adjuvant CRT	0.65	0.57–0.74	<0.001	0.66	0.56–0.77	<0.001

**Fig 1:**
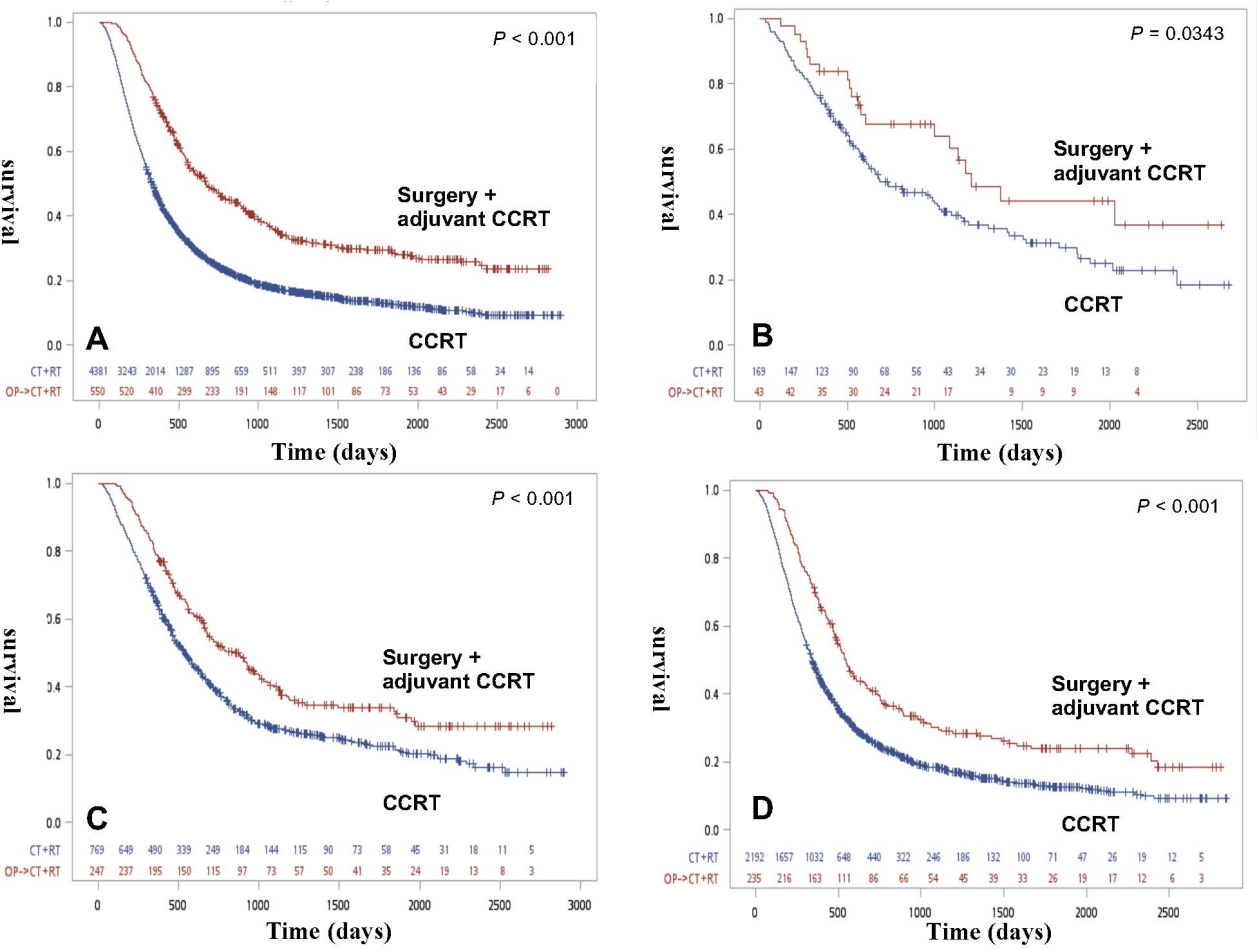
Overall survival of all ESCC patients and each clinical stage before propensity score matching. (A) Kaplan-Meier survival curves for ALL ESCC patients treated via definitive CRT or definitive CRT(p<0.001) (B) Kaplan-Meier survival curves for clinical stage I patients stratified based on treatment modality (p = 0.0343). (C) Kaplan-Meier survival curves for Clinical stage II patients stratified based on treatment modality (p<0.001). (D) Kaplan-Meier survival curves for Clinical stage III patients stratified based on treatment modality (p<0.001).

The OS curve for all ESCC patients according to the clinical stage was stratified based on treatment strategy (Fig 1). The survival curve of all ESCC patients is shown in Fig 1A. All ESCC patients in the surgery with adjuvant CRT group had a significant superior OS rate (p<0.001). The survival curve was assessed according to the clinical stage. The OS rate in the surgery with adjuvant CRT group was better in the definitive CRT in clinical I/II/III patients. (p<0.05) (Fig 1B–1D).

**Fig 2:**
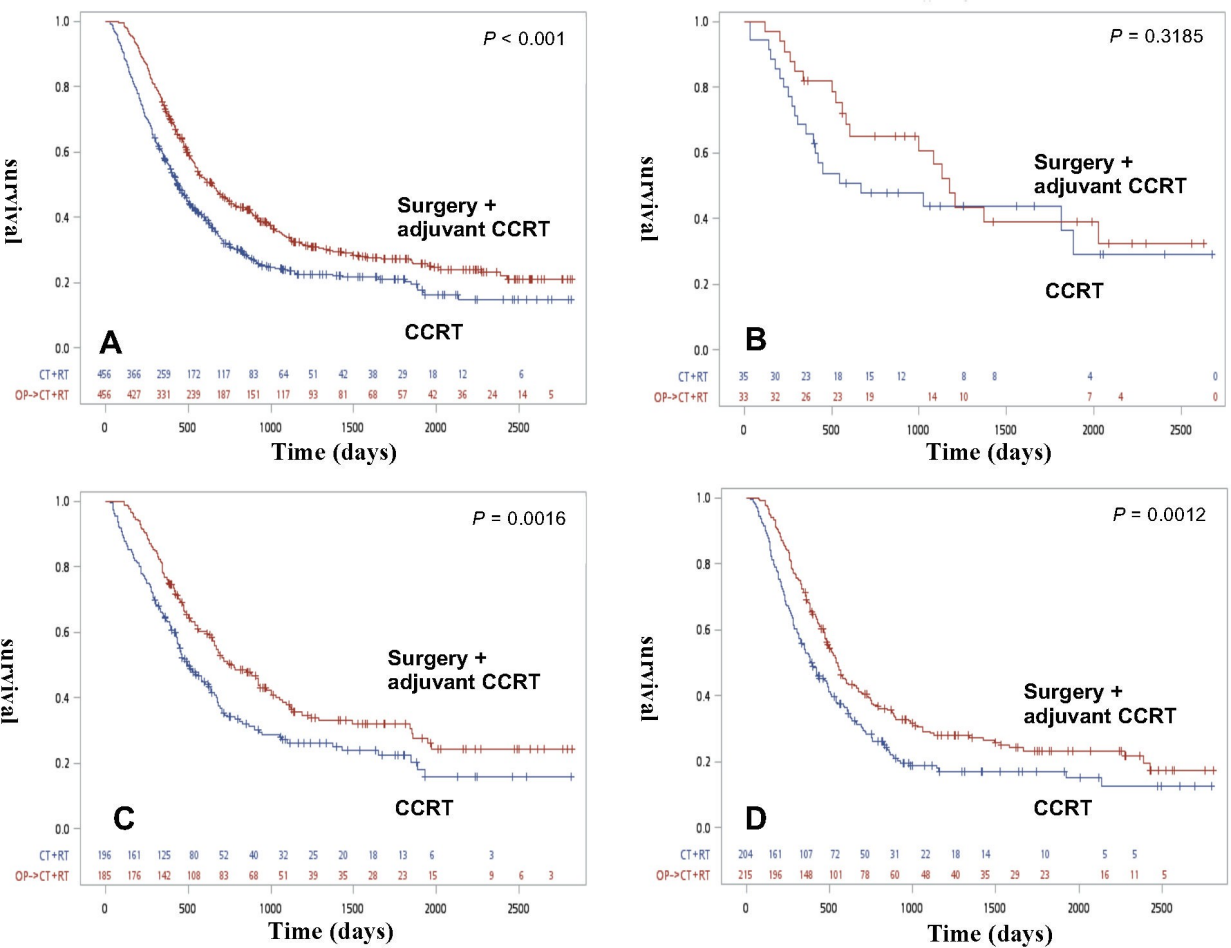
Overall survival of all ESCC patients and each clinical stage after propensity score matching. (A) Kaplan-Meier survival curves for ESCC patients receiving definitive CRT or definitive CRT after propensity matching. (p<0.001). (B) Kaplan-Meier survival curves for clinical stage I patients stratified based on treatment modality after propensity matching. (p = 0.3185). (C) Kaplan-Meier survival curves for clinical stage II patients stratified based on treatment modality after propensity matching (p = 0.0016). (D) Kaplan-Meier survival curves for clinical stage III patients stratified based on treatment modality after propensity matching. (p = 0.0012).

The OS rates for matched patients are shown in Fig 2. ESCC patients in the surgery with CRT group had a significantly better OS outcome than patients in the definitive CRT group (p<0.001) (Fig 2A). The OS curve for all matched patients according to the clinical stage was stratified based on treatment strategy (Fig 2). No significant survival difference was found between the two groups for matched clinical stage I patients (p = 0.3185) (Fig 2B). For clinical stage II and III patients, the surgery with adjuvant CRT group resulted in better OS rates than the definitive CRT group (p<0.001).

## Discussion

The study investigated the OS of patients with clinical stage T1-T3, N0-N3 ESCC who were treated with definitive CRT or surgery with adjuvant CRT in Taiwan. The results demonstrated that Charlson score, clinical T stage, tumor differentiation, tumor length and treatment modality were independent prognostic factors for OS in multivariable analysis after propensity score matching. The results also suggested surgery with adjuvant CRT had better survival than definitive CRT in clinical stage II and III ESCC patients. With regard to clinical stage I ESCC patients, no significant difference in OS was found between the definitive CRT group and the surgery with adjuvant CRT group.

Definitive CRT is recommended as one of the definitive treatments for clinical stage T1b-T4a, N0/N+ ESCC by NCCN guidelines [7]. In Taiwan, almost 46% of ESCC patients received definitive CRT as definitive treatment between 2008 and 2014 [4,9]. The treatment efficacy of definitive CRT was established by some clinical trials, such as the RTOG 85–01 and RTOG 94–05 trials [10,11]. Mikhail et al. reviewed 12 trials and illustrated that 2-year OS of definitive chemotherapy ranged from 18% to 54% and 3-year OS ranged from 22.2% to 64.3% [10,12–17]. This study reported that 2-/3-year OS were 24.8%/17.8% before matching, and 31.9%/23.8% after matching. These survival outcomes were worse than the previous literature possibly because patients who received definitive CRT were diagnosed at advanced stages and were not suitable candidates for surgery. Furthermore, patient preference and hospital facilities may influence the definitive treatment modality. This might explain why definitive CRT could not provide a favorable survival outcome to patients with ESCC. As for clinical stage I ESCC patients, the result were inconclusive due to the small sample size after matching.

Although surgery with adjuvant CRT was not one of the recommended treatments for locally advanced ESCC by NCCN guidelines, more than a few ESCC patients chose it as a definitive treatment in Taiwan [6,9]. For adjuvant CRT, NCCN guidelines suggested only patients with positive surgical margins should receive adjuvant CRT regardless of their nodal status [7]. However, some studies reported that adjuvant CRT could increase OS, time to recurrence, recurrence-free survival and decrease the rates of metastasis and overall recurrence in ESCC [18–21]. Hsu et al. showed that post-operative CRT was associated with longer OS, longer disease-free survival and lower locoregional recurrence. However, Chen et al. illustrated that patients with resectable thoracic ESCC may not benefit from adjuvant CRT [22]. Chen et al. illustrated that 1-year and 2-year OS rates for patients with ESCC who received surgery with adjuvant CRT were 67.5% and 41.4%. In the present study, 1-year and 2-year OS rates for patients with ESCC who received surgery with adjuvant CRT were 74.35% and 47.81% before matching and 72.35% and 45.74% after matching. Our results demonstrated that surgery with adjuvant CRT provided acceptable survival outcome. Usually, the side effects and toxicity of adjuvant CRT are worries for patients who undergo esophagectomy. Before the development of minimally invasive esophagectomy, patients who underwent open esophagectomy were unlikely to cope with any adjuvant therapy. With the advancement of minimally invasive esophagectomy, the early restoration of physical function may make patients able to endure adjuvant treatment [23]. The early recovery after minimally invasive esophagectomy may improve the delivery of adjuvant CRT. Thus, the role of adjuvant CRT in ESCC should be reappraised in the era of minimally invasive esophagectomy.

Neoadjuvant CRT plus esophagectomy and esophagectomy alone were main treatment strategies for clinical stage T1b-T4a, N0/N+ ESCC patients in the NCCN guidelines as well. Neoadjuvant CRT plus esophagectomy is a widely acceptable treatment strategy in the world. The CROSS trial and other systematic reviews reported the treatment efficacy of neoadjuvant CRT plus esophagectomy [24,25]. However, some clinical trials were unable to find out the role of esophagectomy when added to CRT [26,27]. As for esophagectomy alone, multiple studies were conducted to compare surgery alone with definitive CRT [28–30]. These studies concluded that no statistically significant difference in OS between surgery alone and definitive CRT was seen. Therefore, further head-to-head comparison studies are needed to conclude the advantages and disadvantages of each treatment strategy.

In Taiwan, 14,394 patients were newly diagnosed with ESCC between 2008 and 2014. Most of them received definitive CRT, CRT plus esophagectomy, esophagectomy alone, or esophagectomy plus adjuvant CRT [4,9]. In order to find out which treatment strategy could provide the best treatment outcome for patients with locally advanced ESCC, head-to-head comparisons between each treatment strategy were necessary. Wang et al. had compared definitive CRT with neoadjuvant CRT plus esophagectomy, and definitive CRT with esophagectomy alone [4,6]. Until today, there was no literature comparing definitive CRT and esophagectomy plus adjuvant CRT directly. This is the first study to directly compare definitive CRT and esophagectomy plus adjuvant CRT using a large number of cases and propensity score matching to reduce the selection bias.

The TCR database is a multi-center and population-based database. It has limitations that are inherent to all cohort studies because it is a retrospectively maintained database. First of all, the definitive CRT group were tended to be at advanced stage in the present study, hence, propensity score matching was performed to minimize selection bias. There are still some confounding factors that we cannot overcome, and also there was no information about the performance status before surgery and CRT. Second, details about surgical procedures, postoperative complications and recurrence were lacking in this study. Third, the TCR database didn’t include the information about how many of these ESCC patients were planned to treat with esophagectomy and adjuvant CRT from the initial diagnosis and staging. It would be optimal to run a study to compare the four treatment strategies to conclude the best treatment options according to the stages. However, propensity score matching between four treatment groups was not conducted because the overall number of patients in certain treatment groups were too small. Due to the retrospective nature of the study, a randomized control trial to validate results is necessary.

## Conclusions

This propensity-matched study demonstrated that esophagectomy with adjuvant CRT had better survival than definitive CRT in clinical stage II/III ESCC. As for clinical stage I ESCC patients, more data are needed to conduct a convincing conclusion.

## Supporting information

S1 Data (XLSX)
